# Exploring mental health experiences of parents of children with chronic illnesses: Trauma‐related symptoms and isolation

**DOI:** 10.1111/ped.70218

**Published:** 2025-10-04

**Authors:** Madoka Kobayashi, Maoko Hayakawa, Ayaka Ishii‐Takahashi, Kyoko Tanaka

**Affiliations:** ^1^ Department of Psychosocial Medicine National Center for Child Health and Development Tokyo Japan; ^2^ Pediatric Medical and Pediatric Surgery Center Juntendo University Hospital Tokyo Japan; ^3^ Human Developmental Sciences Ochanomizu University Tokyo Japan; ^4^ Department of Psychiatry Tokyo Medical University Tokyo Japan; ^5^ Department of Child Neuropsychiatry The University of Tokyo Hospital Tokyo Japan; ^6^ Department of Pediatrics, Faculty of Medicine Juntendo University Tokyo Japan

**Keywords:** consultation‐liaison psychiatry, CSHCN, medical trauma

Chronic childhood illness is a potentially traumatic experience for children and parents, and prolonged trauma symptoms are said to affect attachment styles, family functioning, and decision‐making in transitions.^1^ However, few reports have examined the details of the content, rate, and natural history of these symptoms. We report here on the prevalence of prolonged stress and trauma reactions in caregivers of children with chronic conditions, and how these reactions are related to feelings of isolation.

With the approval of the Ethics Committee of the National Center for Child Health and Development, we analyzed emotional changes (such as stress and trauma) associated with the care of children with chronic conditions. Parents of children with special health care needs (CSHCN), who provided written consent, were asked to retrospectively compare their current emotional state with how they felt during the first month after their child's diagnosis. The study period was from January 2019 to March 2021. Original questionnaire responses were obtained from 109 participants, 92% of whom were mothers. The ICD‐10 disease classification of the participants' children was relatively broadly distributed, although congenital malformations and chromosomal abnormalities (19 of 109 participants) and renal‐urogenital diseases (17 of 109 participants) were common. In terms of emotional changes in caregivers after diagnosis, as shown in Figure 1, 94% of parents showed some kind of stress reaction during the first month after diagnosis, and 64% of these reactions persisted thereafter. More than half of the parents showed feelings of depression, self‐blame, tension, anxiety, and hopelessness soon after the disease was discovered, but these feelings of depression and hopelessness tended to improve within 1 month. On the other hand, violence, violent words, irritability, and child‐rearing difficulties tended to persist, and after 1 month, new symptoms of poor parental health, anger toward the child, and irritability appeared more frequently. Based on the results of the Parenting Stress Index (62 of 109 participants responded), parenting stress among caregivers of CSHCN is elevated due to both child‐related and parent‐related factors (Figures S1 and S2). On the child's side, factors such as perceiving the child as not meeting expectations or as having problems suggest that how parents perceive and accept their child's chronic condition significantly influences their level of parenting stress (see also Supporting Information).

**FIGURE 1:**
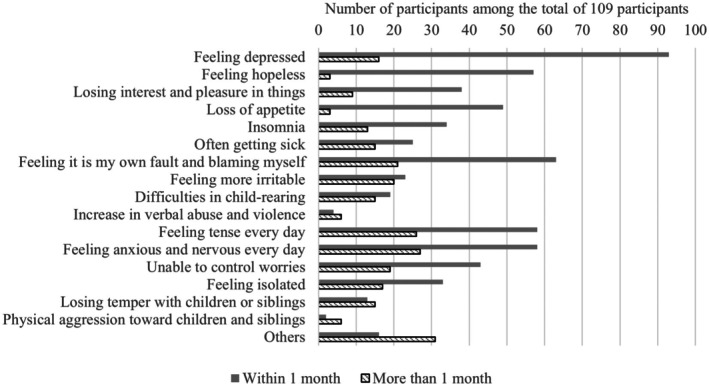
Changes in the parental stress reactions after the child's illness was identified.

From this study, more than half of the caregivers of CSHCN had persistent trauma‐related symptoms. Caregivers with prolonged feelings of isolation also showed more trauma‐related symptoms. In addition, symptoms that were more likely to persist over time, such as violence, verbal abuse, irritability, and difficulty in parenting, and symptoms that were more likely to be recognized over time, such as poor parental health, anger toward the child, and irritability, were more directly related to child rearing. Furthermore, the results showed that more trauma‐related symptoms tended to be observed in caregivers with persistent feelings of isolation, which were related to the sense of isolation. Chronic childhood illness is a high risk for maltreatment, and the American Academy of Pediatrics suggests that it can be reduced by managing economic hardship, family stress, and other long‐term needs, which has been reaffirmed during the COVID‐19 pandemic.^2^, ^3^ Moreover, previous literature suggests that the greater the degree of disability and morbidity complexity, the lower the family resilience and the higher the frequency of family‐related adverse childhood experiences (ACEs).^4^ Previous studies have also suggested that negative perceptions of the child or the child's illness can increase parenting stress, potentially leading to abuse and caregiver health issues.^5^ Caregivers of CSHCN may require parenting support based on a trauma‐informed approach,^6^ which considers the conflicts and experiences associated with raising a child with a chronic illness. We would like to further address the limitations of this study, including the establishment of a control group, examining longitudinal changes, and pursuing a categorical approach by analyzing each condition and analysis that takes into account factors that influence family adjustment and parenting. Furthermore, the inclusion of the COVID‐19 pandemic during the study period may have affected the findings due to restrictions on parental visitation and broader societal impacts.

In conclusion, the high level of trauma reactions and parenting stress among caregivers of CSHCN is noteworthy in terms of the parent–child relationship and ACEs. The experience of COVID‐19, which resulted in deepened social isolation, has led to the recognition that CSHCN are a particularly vulnerable population in terms of mental health, and although family support will continue to be a focus of attention in the care of chronically ill children with physical and emotional burdens, there is a need to understand and support the unique conflicts, burdens, and medical trauma of raising children with chronic illnesses. However, it was inferred from this study that it is necessary to understand the unique struggles and burdens of raising a child with a chronic illness and medical trauma, and to consider how support should be provided.

## AUTHOR CONTRIBUTIONS

Dr. Kobayashi conducted the analysis and wrote the paper based on the research question, which Dr. Tanaka, as the leader of this research project, designed the research plan, implemented it, and summarized the results. Ms. Hayakawa contributed to the study design and interpretation of data. Dr. Ishii‐Takahashi contributed to the conception and the planning of the main AMED project. All authors reviewed and approved the final version of the manuscript.

## INFORMED CONSENT

The research was approved by the ethics committee of the National Center for Child Health and Development. Written consent was obtained from the participants.

## FUNDING INFORMATION

This study was funded by the Japan Agency for Medical Research and Development (AMED) “Research on Mental Health Care for Children with Illness and Their Families: Investigation of the Current Situation Toward Building a Support Program” (PI: Ayaka Ishii‐Takahashi, Co‐PI: Kyoko Tanaka, 2018–2022).

## CONFLICT OF INTEREST STATEMENT

The authors declare no conflicts of interest.

## Supporting information


Figure S1.
