# Supplementary material for: Exploring mental health experiences of parents of children with chronic illnesses: Trauma‐related symptoms and isolation
Source: Pediatr Int. 2025 Oct 4;67(1):e70218. doi: 10.1111/ped.70218 (PMC12506838; doi:10.1111/ped.70218)
Supplement: Supplementary file 1 — Figure S1. [file PED-67-e70218-s001.docx]

Supporting Information

Supplemental Figure 1


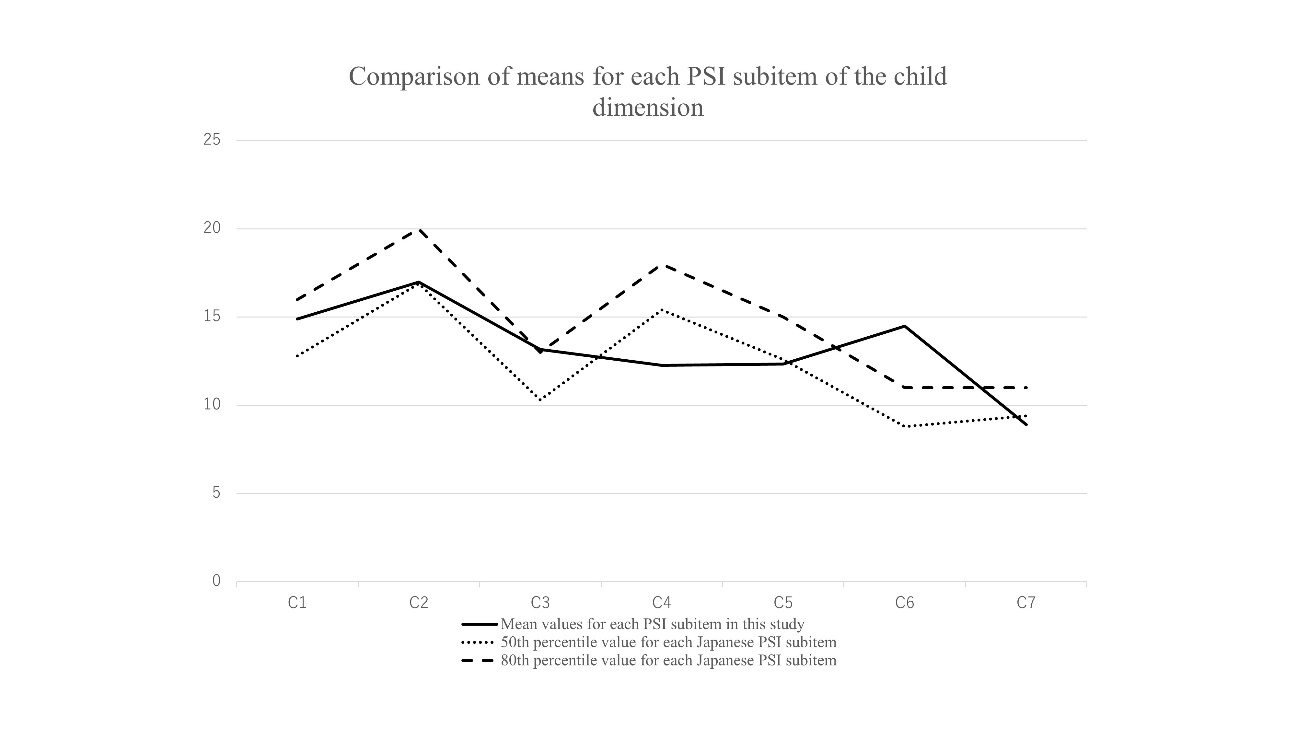


Item Content

C1: Child has few reactions that please parents, C2: Child is in a bad mood, C3: Child does not meet expectations, C4: Child easily distracted/hyperactivity, C5: Child follows parents around or has difficulty getting used to people, C6: Feeling that the child has problems, C7: Child is sensitive to stimuli/Hard to get used to things.

Supplemental Figure 2


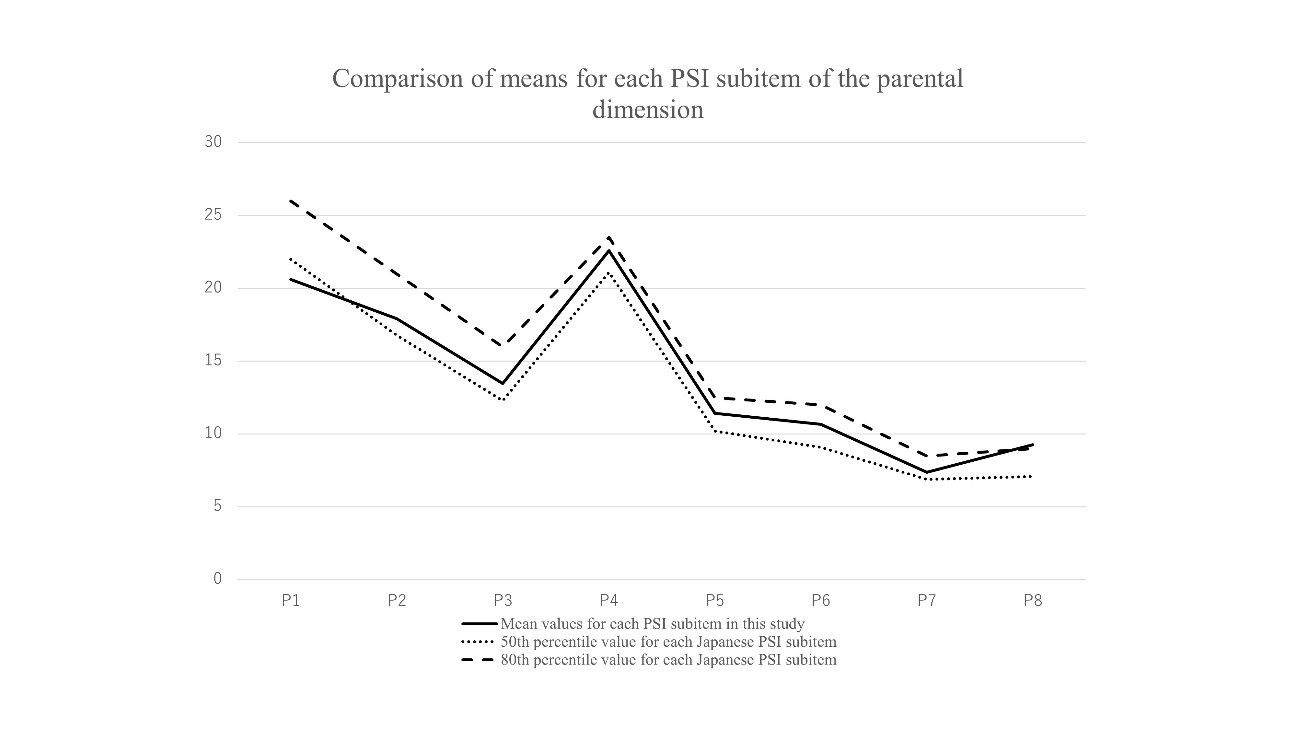


Item Content

P1: Regulations created by parental roles, P2: Social isolation, P3: Marital Relationships, P4: Parental competence, P5: Depression and guilt, P6: Feelings after the child leaves the hospital, P7: Difficulty feeling attachment to child, P8: Health condition.

Figure1,2 legends

The mean values in this study were around the 70th percentile for both child-related and parent-related characteristics, indicating a higher tendency. Notably, “Child does not meet expectations” and “Feeling that the child has problems” were above the 80th percentile, as was parental “Health condition”.
